# Analysis of *SLC26A4, FOXI1,* and *KCNJ10* Gene Variants in Patients with Incomplete Partition of the Cochlea and Enlarged Vestibular Aqueduct (EVA) Anomalies

**DOI:** 10.3390/ijms232315372

**Published:** 2022-12-06

**Authors:** Leonid A. Klarov, Vera G. Pshennikova, Georgii P. Romanov, Aleksandra M. Cherdonova, Aisen V. Solovyev, Fedor M. Teryutin, Nikolay V. Luginov, Petr M. Kotlyarov, Nikolay A. Barashkov

**Affiliations:** 1 Yakut Science Centre of Complex Medical Problems, 677000 Yakutsk, Russia; 2 Republican Hospital # 1—National Center of Medicine, 677019 Yakutsk, Russia; 3 Laboratory of Molecular Biology, M.K. Ammosov North-Eastern Federal University, 677027 Yakutsk, Russia; 4 Russian Scientific Center for Radiology, 117997 Moscow, Russia

**Keywords:** *SLC26A4*, *FOXI1*, *KCNJ10* genes, hearing loss, audiometric examination, computer tomography, inner ear anomalies, incomplete partition type 1 (IP-1), incomplete partition type 2 (IP-2), enlarged vestibular aqueduct (EVA), genotype-phenotype analysis, DFNB4, Pendred syndrome, Eastern Siberia, Russia

## Abstract

Pathogenic variants in the *SLC26A4*, *FOXI1*, and *KCNJ10* genes are associated with hearing loss (HL) and specific inner ear abnormalities (DFNB4). In the present study, phenotype analyses, including clinical data collection, computed tomography (CT), and audiometric examination, were performed on deaf individuals from the Sakha Republic of Russia (Eastern Siberia). In cases with cochleovestibular malformations, molecular genetic analysis of the coding regions of the *SLC26A4*, *FOXI1*, and *KCNJ10* genes associated with DFNB4 was completed. In six of the 165 patients (3.6%), CT scans revealed an incomplete partition of the cochlea (IP-1 and IP-2), in isolation or combined with an enlarged vestibular aqueduct (EVA) anomaly. Sequencing of the *SLC26A4*, *FOXI1*, and *KCNJ10* genes was performed in these six patients. In the *SLC26A4* gene, we identified four variants, namely c.85G>C p.(Glu29Gln), c.757A>G p.(Ile253Val), c.2027T>A p.(Leu676Gln), and c.2089+1G>A (IVS18+1G>A), which are known as pathogenic, as well as c.441G>A p.(Met147Ile), reported previously as a variant with uncertain significance. Using the AlphaFold algorithm, we found in silico evidence of the pathogenicity of this variant. We did not find any causative variants in the *FOXI1* and *KCNJ10* genes, nor did we find any evidence of digenic inheritance associated with double heterozygosity for these genes with monoallelic *SLC26A4* variants. The contribution of biallelic *SLC26A4* variants in patients with IP-1, IP-2, IP-2+EVA, and isolated EVA was 66.7% (DFNB4 in three patients, Pendred syndrome in one patient). Seventy-five percent of *SLC26A4*-biallelic patients had severe or profound HL. The morphology of the inner ear anomalies demonstrated that, among *SLC26A4*-biallelic patients, all types of incomplete partition of the cochlea are possible, from IP-1 and IP-2, to a normal cochlea. However, the dominant type of anomaly was IP-2+EVA (50.0%). This finding is very important for cochlear implantation, since the IP-2 anomaly does not have an increased risk of “gushers” and recurrent meningitis.

## 1. Introduction

Autosomal recessive deafness Type 4 (DFNB4, OMIM #600791) is a hereditary disease, characterized by sensorineural hearing loss (HL), in isolation or combined with specific inner ear abnormalities [1,2,3,4,5,6] such as an enlarged vestibular aqueduct (EVA) and cystic cochlear abnormalities [7,8], which were previously known as Mondini malformations [7]. Presently, on the basis of the radiographic classifications proposed by Sennaroglu (2002, 2017), cystic cochlear anomalies are differentiated into Incomplete Partition Type 1 (IP-1) and Incomplete Partition Type 2 (IP-2) [9,10]. At present, the term “Mondini” is used only if a triad of malformations is present (IP-2 + a minimally dilated vestibule + an enlarged vestibular aqueduct) [10]. Currently, pathogenic variants in the *SLC26A4*, FOXI1, and *KCNJ10* genes are responsible for DFNB4 (OMIM #600791). Moreover, pathogenic variants in the *SLC26A4* gene are associated with Pendred syndrome (PS, OMIM #274600), in which sensorineural HL is combined with thyroid dysfunction. The *SLC26A4* gene is located on chromosome 7q22–q31, contains 21 exons, and encodes the transmembrane transporter protein known as pendrin (PDS/SLC26A4) [11,12,13,14,15]. Pendrin is a multi-transmembrane (TM) protein composed of 780 amino acids, consisting of 12–14 TM segments and a segment of the intracellular STAS (Sulfate Transporter and Anti-Sigma factor antagonist) functional domain [16,17]. The spectrum and frequency of the *SLC26A4* variants varies in different populations worldwide [18,19,20,21,22,23,24,25,26,27,28,29,30,31,32,33,34,35]. The highest prevalence of *SLC26A4* gene pathogenic variants was found in populations from East Asia (Mongolia, China, Taiwan, Japan, and Republic of Korea) [18,19,20,21,22,23,24,25,26,27,28]. Over 65–95% of East Asian patients with EVA have biallelic pathogenic variants in the SLC26A4 gene [18,19,20,21,22,23,24,25,26,27,28]. The high prevalence of some *SLC26A4* variants, such as c.2168A>G p.(His723Arg) and c.919-2A>G (IVS7-2A>G) in East Asia, can be explained by the founder effect [18]. The prevalence of the pathogenic *SLC26A4* variants in Europe and North America is much lower. Approximately 25–50% of patients with EVA from North America and Europe have biallelic pathogenic variants in the *SLC26A4* gene [2,6,18,29,30,31,32,33,34]. The low frequency of the *SLC26A4* variants in western Eurasian patients compared with eastern Eurasian patients is probably influenced by different genetic backgrounds [24,30,35,36]. Moreover, some authors have explained this distinction using the hypothesis of the CEVA haplotype [37]. The CEVA haplotype was recently identified through massive parallel sequencing and was found in the trans-position with high frequency among *SLC26A4*-monoallelic patients of Caucasian origin [37]. Five out of the seven nucleotides of the CEVA haplotype (TGTTCGA, underlined) match with the consensus binding sequence reported for *FOXI1*, which may affect the expression of *SLC26A4* [37].

In 2007, Yang et al. [38] described two DFNB4 families with heterozygous variants in the *FOXI1* gene and proposed that this gene showed significantly decreased luciferase activation in promoter-reporter assays, suggesting that this variant compromised the ability of *FOXI1* to transactivate SLC26A4 and was causally related to disease [38,39,40,41]. The *FOXI1* gene is located on chromosome 5, contains two coding exons, and belongs to the forkhead family of transcription factors, characterized by a distinct forkhead domain. This gene may play an important role in the development of the cochlea and vestibulum, as well as in embryogenesis [42,43,44]. In addition, the encoded protein has been found to be required for the transcription of four subunits of a proton pump found in the inner ear, the kidney, and the epididymis [45,46,47]. Moreover, Yang et al. proposed the hypothesis of digenic inheritance in patients with monoallelic *SLC26A4* variants associated with the heterozygous variants of the *FOXI1* and *KCNJ10* genes [38,47,48,49]. The *KCNJ10* gene is located on chromosome 1, contains one coding exon, and encodes a member of the inward rectifier-type potassium channel family, characterized by having a greater tendency to allow potassium to flow into, rather than out of, a cell [42,43,44]. The encoded protein may form a heterodimer with another potassium channel protein and may be responsible for the potassium buffering action of glial cells in the brain [42,43,44]. The OMIM database describes three family cases (https://omim.org/entry/600791, accessed on 2 December 2022) with digenic inheritance in patients with monoallelic *SLC26A4* variants associated with heterozygous variants of the *FOXI1* and *KCNJ10* genes.

In Russia, the *FOXI1* and *KCNJ10* genes have not previously been analyzed among patients with HL. However, pathogenic variant analysis of the *SLC26A4* gene was previously performed in 246 families with HL cases from the Bashkortostan Republic of Russia (Volga–Ural Region) [50] and in 20 patients with Pendred syndrome, EVA, and/or Mondini anomalies in a geographically dispersed sample [51]. Analyses of pathogenic variants of the *SLC26A4* gene in 313 patients with HL in the Tyva and Altai Republics of Russia (Southern Siberia) revealed the different contributions of biallelic *SLC26A4* pathogenic variants to the etiology of HL (28.2% in Tuvinian and 4.3% in Altaian patients) [52]. In total, 14 recessive pathogenic variants of the *SLC26A4* gene were identified: c.85G>C p.(Glu29Gln), c.l49T>G p.(Leu50Arg), c.170C>A p.(Ser57Ter), c.222G>T p.(Trp74Cys), c.317C>A p.(Ala106Asp), c.919-2A>G (IVS7-2A>G), c.1001G>T p.(Glu334Val), c.1003T>C p.(Phe335Leu), c.1229C>T p.(Thr410Met), c.1790T>C p.(Leu597Ser), c.1545T>G p.(Phe515Leu), c.2027T>A p.(Leu676Gln), c.2034+1G>A (a splice site variant), and c.2168A>G p.(His723Arg) [50,51,52].

In the present study, consecutive genotype–phenotype analyses were performed in 165 deaf individuals from the Sakha Republic of Russia (Eastern Siberia), including clinical data collection, computed tomography, audiometric examination, and Sanger sequencing of the coding regions of the *SLC26A4*, FOXI1, and *KCNJ10* genes associated with DFNB4.

## 2. Results

### 2.1. Allelic Frequency and Clinical Significance of the Identified Variants in the SLC26A4, FOXI1, and KCNJ10 Genes

In six of the 165 patients (3.6%), CT scans revealed an incomplete partition of the cochlea (IP-1 and IP-2) in isolation or combined with an enlarged vestibular aqueduct (EVA) anomaly. In patients with incomplete partition of the cochlea (IP-1 and IP-2) and/or an enlarged vestibular aqueduct (EVA) anomaly, we sequenced the coding region of the *SLC26A4*, *FOXI1*, and *KCNJ10* genes. In the *SLC26A4* gene, five previously known variants were found, four of which were missense variants, namely c.85G>C p.(Glu29Gln) (10%), c.441G>A p.(Met147Ile) (20%), c.757A>G p.(Ile253Val) (20%), and c.2027T>A p.(Leu676Gln) (20%), with one variant that affected the donor splicing site c.2089+1G>A (IVS18+1G>A) (10%). Two synonymous variants were found in the *FOXI1* gene: c.279G>A p.(Arg93=) (30%) and c.1044T>C p.(Tyr348=) (90%), and one previously known missense variant, c.811C>T p.(Arg271Cys) (10%), was found in the *KCNJ10* gene. The allelic frequency of the *SLC26A4*, *FOXI1*, and *KCNJ10* gene variants identified in this study is presented in Table 1. The allelic frequency of the identified variants in some populations according to gnomAD data is shown in Table S1. Among the variants present in the ClinVar database, the three *SLC26A4* variants c.85G>C p.(Glu29Gln), c.2027T>A p.(Leu676Gln), and c.2089+1G>A (IVS18+1G>A) are considered to be pathogenic. One variant, c.757A>G p.(Ile253Val), was absent in ClinVar; however, it was described as likely pathogenic in the Deafness Variation Database (https://deafnessvariationdatabase.org/ accessed on 28 September 2022) (Table 1). The two *FOXI1* variants c.279G>A p.(Arg93=) and c.1044T>C p.(Tyr348=), and one *KCNJ10* variant c.811C>T p.(Arg271Cys), were classified as benign or likely benign (Table 1). The c.441G>A p.(Met147Ile) variant of the *SLC26A4* gene has conflicting interpretations of pathogenicity: in ClinVar, it is listed as likely benign (1) and of uncertain significance (6) (https://www.ncbi.nlm.nih.gov/clinvar/variation/229258/ accessed on 28 September 2022) (Table 1).

### 2.2. In Silico Assessment of the Pathogenic Effect of the c.441G>A p.(Met147Ile) Missense Variant of the SLC26A4 Gene on the Function and/or Structure of Pendrin (SLC26A4)

In the HGMD database (http://www.hgmd.cf.ac.uk/ac/all.php accessed on 28 September 2022), the c.441G>A p.(Met147Ile) variant of the *SLC26A4* gene is associated with Pendred syndrome and enlarged vestibular aqueduct (EVA), as published by Jonard et al. (2010) [53]. However, the authors identified only heterozygous variants in patients presenting unilateral HL and ipsilateral EVA [53], which is not the cause of disease with the autosomal recessive type of inheritance. According to the ACMG guidelines, a variant of uncertain significance (VUS) should not be used in clinical decision-making [54,55]. Regardless of the results of in silico pathogenicity prediction programs (SIFT, Polyphen-2, PROVEAN, and Pathogenic variant Taster), which all rated it as damaging (Table S2), we applied an additional in silico pathogenicity assessment of this variant; since, in the most programs, computational prediction methods are based on a similar algorithm of evolutionary conservatism. However, the effect of a missense change depends, not only on the evolutionary conservation of an amino acid or nucleotide, but also on other criteria, such as the location and context within the protein sequence, and the biochemical consequence of the amino acid substitution [54]. However, the crystal structure of human pendrin protein is currently unknown, making a predictive analysis of the pathogenic effect of *SLC26A4*-variants on the function and/or structure of the pendrin protein impossible [16].

In this connection, using the AlphaFold 2.0 AI system [56] and the PyMOL molecular graphics system [57], we modeled and aligned the mutant and native protein structures, to evaluate the likely pathogenic effect on the function and/or structure of pendrin (SLC26A4) (Figures S1 and S2). A detailed in silico analysis and a discussion of the results are presented in the Supplementary Materials. As a result, the calculated similarity index of the two structures’ RMSD (root mean square deviation of the atomic positions) was less than 2 Å, which indicates that the missense p.(Met147Ile) substitution theoretically does not violate the structural integrity of the SLC26A4 protein (Figure S2). Probably, the pathogenic effect of this variant occurs at the functional level, since the analyzed p.(Met147Ile) substitution is located in a critical region of the core domain (Figures S3 and S4), the violation of which can lead to incorrect substrate transport or the appearance of toxic conformations. Thus, the obtained data indicate that the c.441G>A p.(Met147Ile) variant of the *SLC26A4* gene, which is located in an evolutionarily conservative region (the α3-helix region of the TMD) (Figure S5), can lead to the manifestation of diseases associated with a functional disruption of the core domain of the pendrin protein (SLC26A4). Three other missense pathogenic variants of the *SLC26A4* gene are known in this evolutionarily conserved region that lead to methionine substitution at the 147 amino acid position of pendrin: c.439A>C p.(Met147Leu), c.439A>G p.(Met147Val), and c.440T>C p.(Met147Thr) (Table S3). This finding provides additional evidence of the pathogenicity of the c.441G>A p.(Met147Ile) variant of the *SLC26A4* gene. In accordance with the criteria recommended by ACMG [54], adapted for hereditary forms of HL [55], the c.441G>A p.(Met147Ile) variant was classified by us as “likely pathogenic” (Table S4).

### 2.3. Phenotypes of Families with Biallelic Variants in the SLC26A4 Gene

Considering the pathological effect of the identified variants, we present the clinical features of three families with biallelic, and probably causative, variants identified in the *SLC26A4* gene.

Family 1

**Figure 1:**
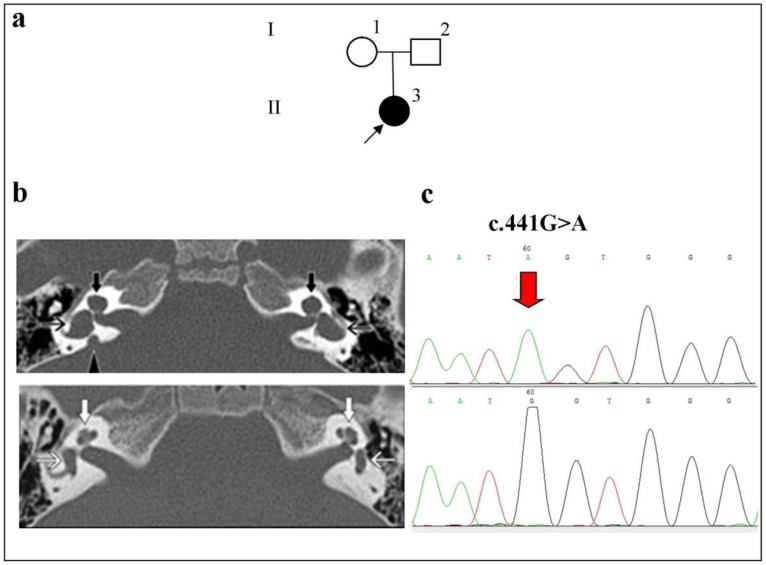
Identification of the c.441G>A p.(Met147Ile) variant in the *SLC26A4* gene in a homozygous state in a patient with bilateral IP-1 and unilateral EVA. **Note.** (**a**) Pedigree of the patient (an arrow indicates the proband). (**b**) Computed tomography (CT) of the temporal bones in the axial projection. Upper panel: Patient II:3 with an IP-1 anomaly (black filled arrows indicate the cystic cochlea), vestibule dilatation (black open arrows), and unilateral EVA (the triangle indicates the enlarged vestibular aqueduct); lower panel, a patient without anomalies (white arrows). (**c**) Upper panel: a fragment of the chromatogram of the *SLC26A4* gene sequence with-the identified c.441G>A p.(Met147Thr) variant in a homozygous state; lower panel: the normal sequence.

Patient II:3 had congenital HL and was five years old, female, and Russian. In this patient, the homozygous c.441G>A p.(Met147Ile) variant was detected in the *SLC26A4* gene (Figure 1).

This is the first report on the identification of the c.441G>A p.(Met147Ile) variant in the *SLC26A4* gene in the homozygous state. Bilateral IP-1 on both sides, bilateral vestibule dilatation, and unilateral EVA were found in her CT scans (Figure 1). The audiological examination revealed severe bilateral HL.

Family 2

**Figure 2:**
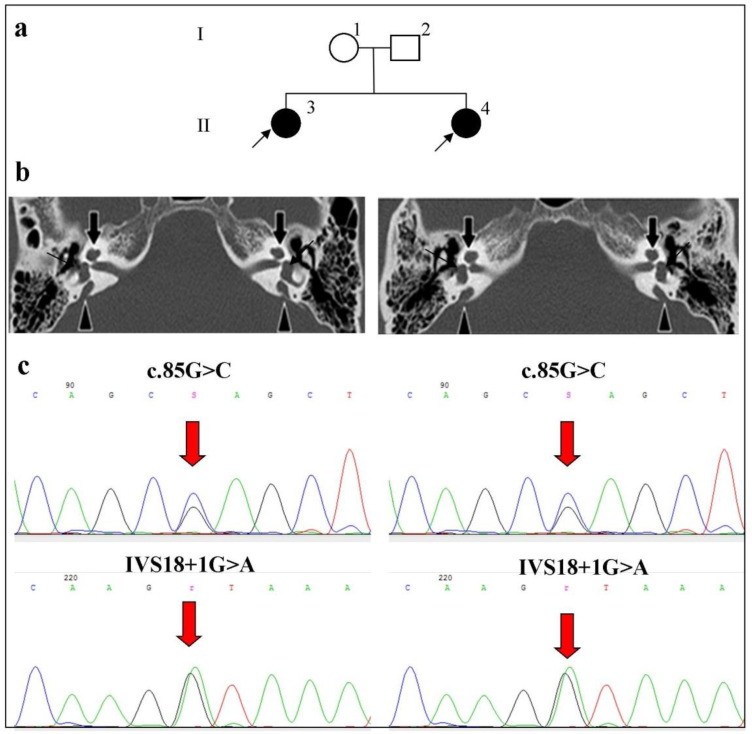
Identification of the c.85G>C p.(Glu29Gln) and c.2089+1G>A (IVS18+1G>A) variants in the *SLC26A4* gene in the compound heterozygous state in siblings with bilateral IP-2 and EVA anomalies. Note. (**a**) Pedigree of the patients (individuals with HL are highlighted in black; arrows indicate probands). (**b**) Computed tomography (CT) of the temporal bones in the axial projection of Patients II:3 and II:4 with IP-2 and EVA (black filled arrows indicate the cystic cochlea; triangles indicate the enlarged vestibular aqueduct); (**c**) The fragment of the chromatogram of the *SLC26A4* gene sequence with the c.85G>C p.(Glu29Gln) and c.2089+1G>A (IVS18+1G>A) pathogenic variants identified in the compound heterozygous state.

Patients II:3 and II:4 (Figure 2) were female siblings, who were 38 and 32 years old and Russian. Sanger sequencing of 21 exons of the *SLC26A4* gene in both sisters revealed the c.2089+1G>A (IVS18+1G>A) (Intron 18) and c.85G>C p.(Glu29Gln) (Exon 2) variants in the compound heterozygous state (Figure 2). CT scans of both sisters revealed an IP-2 anomaly of the inner ear, represented by a cystic cochlea, a dilatated vestibule, and an enlarged vestibular aqueduct (EVA) on both sides (Figure 2). In addition, the observed audiological examination revealed profound bilateral HL in both sisters. No thyroid dysfunction was found in either sister. However, one of the sisters (Patient II:3) had some vestibular impairment and complained of dizziness.

Family 3

**Figure 3:**
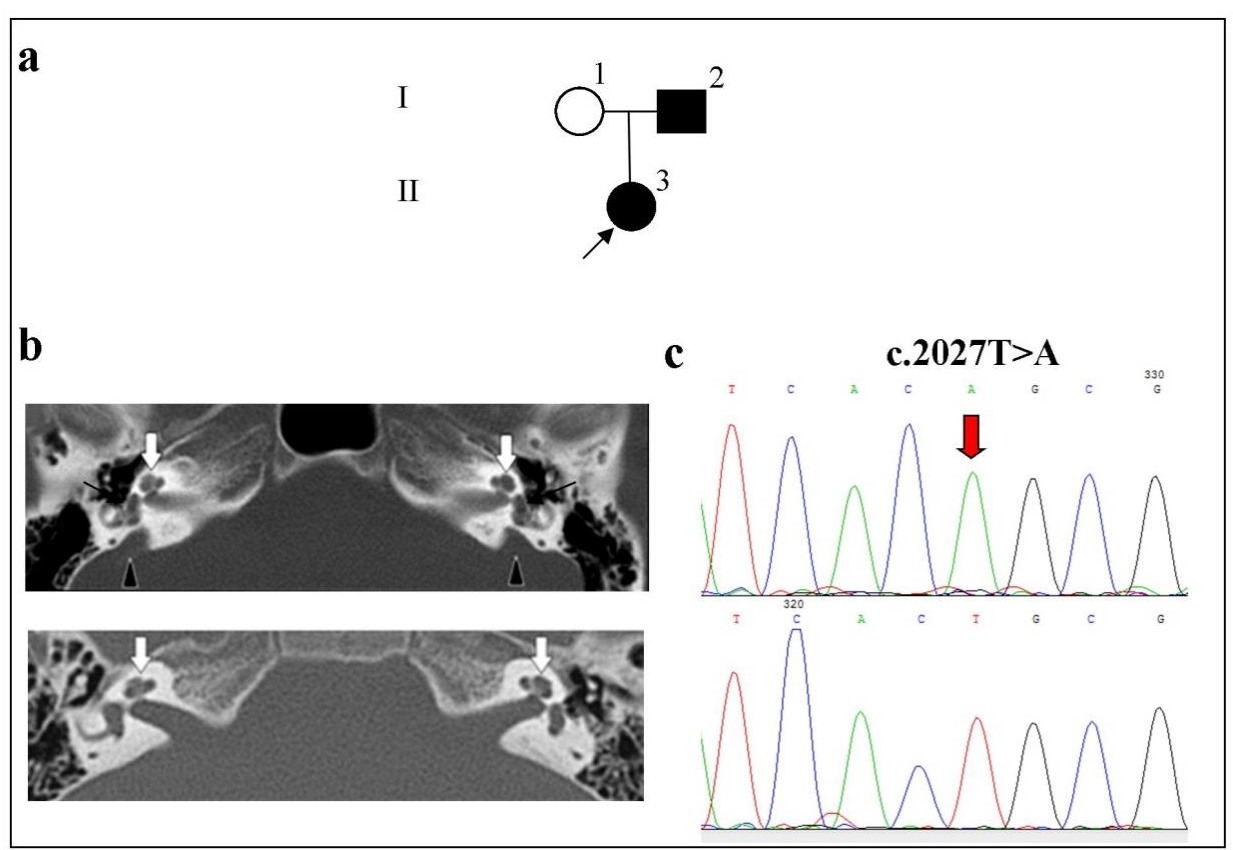
Identification of the c.2027T>A p.(Leu676Gln) variant in the *SLC26A4* gene in a homozygous state in a patient with bilateral EVA anomalies. **Note.** (**a**) Pedigree of the patient (the arrow indicates the proband). (**b**) Computed tomography (CT) of the temporal bones in the axial projection. Upper panel: Patient II:3 with a preserved cochlea (white arrows), vestibule dilatation, and bilateral EVA anomalies (triangles indicate the enlarged vestibular aqueduct); lower panel, a patient without anomalies (white arrows). (**c**) Upper panel: a fragment of the chromatogram of the *SLC26A4* gene sequence with the c.2027T>A p.(Leu676Gln) variant in a homozygous state; lower panel: the normal sequence.

Patient II:3 had congenital HL and was 33 years old, female, and Buryat. The audiological examination revealed bilateral moderate HL. According to the tomograms, the patient had an enlarged vestibular aqueduct (EVA) on both sides. There was a deepening of the contour of the temporal bone in the cerebellopontine angle, caused by the stretching of the sac of the vestibule aqueduct (Figure 3). The patient underwent surgery on the thyroid gland for nodular goiter. In this patient, the homozygous c.2027T>A p.(Leu676Gln) variant was detected in the *SLC26A4* gene (Figure 3).

### 2.4. Clinical and Molecular Genetic Characteristics of Patients with the Studied Inner Ear Malformations

In total, the majority of patients with biallelic pathogenic variants in the *SLC26A4* gene (patient codes 16, 17, and 192) had congenital severe or profound HL (75%), except for a patient with bilateral isolated EVA anomalies (patient code 1091), in whom we registered moderate HL (25%) (Table 2). On the basis of the physical examination and analysis of the available clinical data of the patients with biallelic pathogenic variants in the *SLC26A4* gene, we can conclude that the majority of them (75%) did not complain of episodic vertigo, clumsiness, and vomiting, which could be regarded as vestibular dysfunctions (Table 2) [58,59]. However, one patient with the c.2089+1G>A (IVS18+1G>A) and c.85G>C p.(Gly29Gln) variants in the compound heterozygous state (patient code 16) complained of vertigo (25%) (Table 2). In this patient, we found the symptom triad of Mondini malformation, including a dilated vestibule merging into semicircular canals. A dilated vestibule merging into semicircular canals can be associated with vertigo. However, a similar Mondini malformation was found in her sister, who did not have vertigo. In this case, more in-depth observations were required. An analysis of thyroid dysfunction was carried out, by examining the patients via palpation, without involving other methods of examination (thyroid hormone levels, ultrasound, and perchlorate tests) [59,60]. Most patients with biallelic pathogenic variants in the *SLC26A4* gene (75%) showed no signs of goiter. However, one patient (patient code 1091) with a homozygous c.2027T>A p.(Leu676Gln) pathogenic variant in the *SLC26A4* gene (Table 2) underwent surgery on the thyroid gland for nodular goiter (25%). Thus, taking the identified phenotypic features and data of the molecular genetic analysis together, DFNB4 (OMIM #600791) was confirmed in three patients (patient codes 16, 17, and 192) (Table 2). Pendred’s syndrome (PDS, OMIM #274600) was confirmed in one patient (autosomal recessive HL with bilateral EVA combined with nodular goiter; patient code 1091) with a homozygous variant c.2027T>A p.(Leu676Gln) in the *SLC26A4* gene (Table 2).

In two patients (patient codes 1095, 18), in the *SLC26A4* gene, we found the c.757A>G p.(Ile253Val) variant in the single-heterozygous state, which was previously reported as likely pathogenic (Table 2). Since we did not find a second mutant allele in this gene and did not find any causative variants in the *FOXI1* and *KCNJ10* genes, we cannot relate this monoallelic *SLC26A4*-variant to the DFNB4 phenotype.

## 3. Discussion

In this study computed tomography of the temporal bones revealed IP-1, IP-2, IP-2+EVA, and isolated EVA anomalies in six of the 165 studied patients (3.6%). Among these six patients, Sanger sequencing revealed pathogenic biallelic *SLC26A4*-variants in four patients (4/6), monoallelic *SLC26A4*-variants in two patients (2/6), but no causative variants in *FOXI1* (0/6) and *KCNJ10* (0/6). The total contribution of biallelic (homozygous or compound-heterozygous) pathogenic variants in the *SLC26A4* gene among patients with temporal bone anomalies was 66.7%. On the basis of the obtained clinical and molecular genetic data, DFNB4 was confirmed in three patients, and Pendred’s syndrome was confirmed in one patient. The rate of the biallelic *SLC26A4*-variants in our study was slightly higher than in certain European studies (25–50%) [2,6,18,29,30,31,32,33,34]. Probably this was due to the relatively low rate of monoallelic variants of the *SLC26A4* gene in our sample. The monoallelic *SLC26A4* cases (33.3%) were detected in patients of Siberian origin (two single-heterozygotes for c.757A>G p.(Ile253Val) variant) (Table 2). Since, in this cohort of patients, we did not find a second mutant allele in this gene and did not find any causative variants in the *FOXI1* and *KCNJ10* genes, we supposed that in these *SLC26A4*-monoallelic patients, there are other pathogenic variants in the *trans*-position in the regulator region of this gene, similar to the hypothesis of the CEVA-haplotype [37].

**Figure 4:**
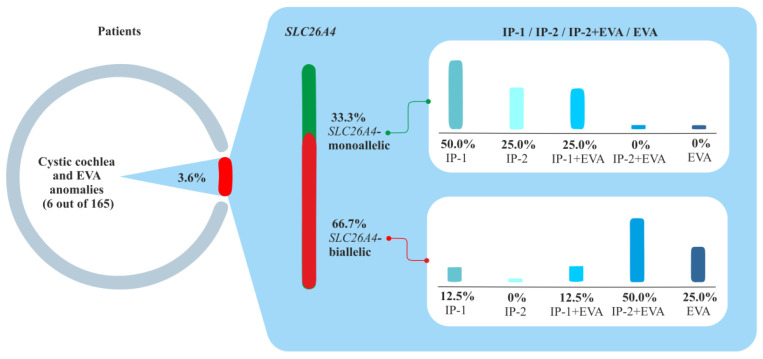
Morphology of the inner ear anomalies in biallelic *SLC26A4* and monoallelic *SLC26A4* patients. **Note.** IP-1, incomplete partition Type 1; IP-2, incomplete partition Type 2; EVA, enlargement of the vestibular aqueduct.

Interestingly, in line with the radiographic classification proposed by Sennaroglu (2002, 2017) [9,10], we found some differences in morphology of the inner ear anomalies between *SLC26A4*-monoallelic and *SLC26A4*-biallelic patients. Among the monoallelic *SLC26A4* patients, the most common type of anomaly was IP-1 without EVA (50%). On the contrary, among the biallelic *SLC26A4* patients, the dominant type of anomaly was IP-2 with EVA (50.0%) (Figure 4).

**Figure 5:**
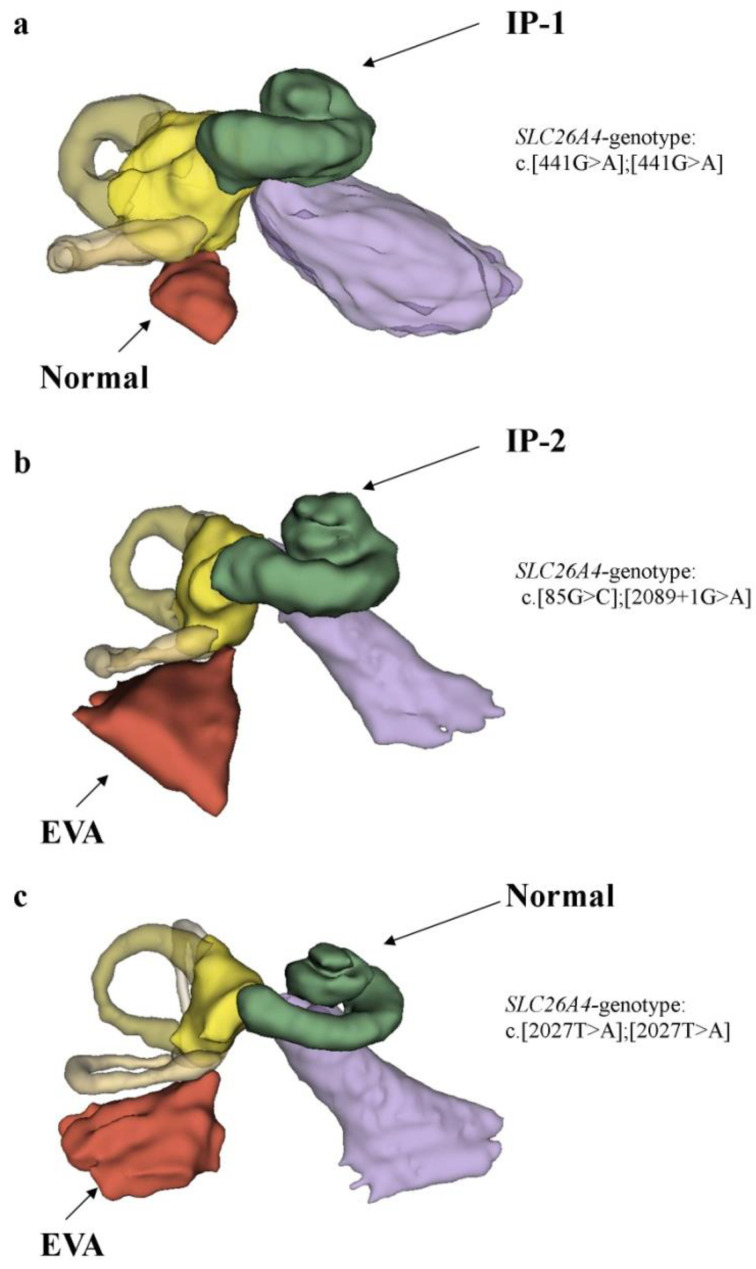
A 3D reconstruction of the incomplete partitions of the cochlea and enlarged vestibular aqueduct anomalies in biallelic *SLC26A4* patients. **Note.** (**a**) Biallelic *SLC26A4* patient with an incomplete partition Type 1 (IP-1) cystic cochlear anomaly; (**b**) biallelic *SLC26A4* patient with an incomplete partition Type 2 (IP-2) cystic cochlear anomaly combined with EVA; (**c**) biallelic *SLC26A4* patient with a normal cochlea and an isolated EVA anomaly.

Our results are consistent with the published data [61]. Previously, Mey et al., in genotype–phenotype analyses of a large cohort of patients with nonsyndromic EVA, described the morphology of inner ear anomalies and found that, among patients with biallelic pathogenic variants in the *SLC26A4* gene (87%), the predominant type of anomaly was IP-2+EVA (including cochlear anomalies, not only isolated EVA) [61]. A study by Mey et al. [61] indicated that the morphology of the inner ear of patients with biallelic *SLC26A4* variants supported the pressure theory proposed by Sennaroglu (2002, 2017) [9,10], allowing for a graded range of severity, ranging from the detectable displacement of the topmost interscalar partitions to a nearly normal cochlea [10]. Our study also supports this hypothesis, because among the biallelic *SLC26A4* patients, we found all types of incomplete partition of the cochlea, from IP-1 and IP-2 to a normal cochlea (Figure 5). Interestingly, in one biallelic *SLC26A4* patient, we found IP-1 on one side and IP-1+EVA on the other side (Figure 2). In a related study by Mey et al. (2019) [61], the IP-1 malformation was not detected in a large cohort of biallelic *SLC26A4* patients with retrievable images of their inner ear morphology. Our study is the first report about these types of anomalies in biallelic *SLC26A4* patients.

Considering that both types of cochlear anomalies are possible, radiographic differences between IP-1 and IP-2 cochlear anomalies in patients with biallelic *SLC26A4* pathogenic variants are significant for the surgery stage of cochlear implantation. The clinical importance of the differences between incomplete partition anomalies is that the IP-2 malformation does not have an increased risk of a “gusher” from the cochleostoma during cochlear implantation, which earlier embryological (IP-1) malformations may have [61]. In addition, patients with IP-2 have a lower risk of recurrent meningitis than patients with IP-1. This is due to the defective stapes footplate and cerebrospinal fluid filling the cochlea. There is a cystic structure in the stapes footplate, which is easily infected during an attack of otitis media. This is very typical for IP-1 but not for IP-2 [10].

## 4. Materials and Methods

### 4.1. Patients

Data on 165 patients with HL were obtained from Republican Hospital No. 1—National Center of Medicine (Yakutsk, Russia), and Republican Hospital No. 2—Center for Emergency Medical Care (Yakutsk, Russia). All patients were examined by an audiologist (collection of complaints, anamnesis of life and illness, ENT examinations (otoscopic, rhinoscopic, and pharyngoscopic studies), acumetry, and sound reactivity testing) and a geneticist (hereditary load, type of inheritance, different syndromic and combined forms). Pediatric patients were additionally examined by a teacher of the deaf (assessment of speech status), a psychoneurologist (neurological status), an endocrinologist (skin, mucous membrane, palpation of the thyroid gland), and a cardiologist (auscultation of the heart). All the participants gave written informed consent to participate in the study. This study was approved by the local Biomedical Ethics Committee of Republican Hospital #2 (Protocol 2, Decision 2 of 24 December 2015).

### 4.2. Audiological Examination

Audiograms of the patients demonstrated variability in bilateral sensorineural HL. In most cases, the hearing thresholds were assessed by pure-tone audiometry, using a clinical tonal audiometer GSI61 (Grason-Stadler Inc., Eden Prairie, MN, USA) in a soundproof room according to the current clinical standards. Air conduction thresholds were obtained at 0.125, 0.25, 0.5, 1, 2, 4, and 8 kHz. The severity of hearing loss was defined as mild (25–40 dB), moderate (41–70 dB), severe (71–90 dB), or profound (above 90 dB).

### 4.3. Computed Tomography of the Temporal Bone

The pyramid of the temporal bone was examined on a 4 slice Somatom Sensation 4 CT scanner (Siemens, Germany) and a 64 slice Somatom Definition AS CT scanner (Siemens, Germany) in axial projection with a tomographic layer thickness of 1 mm, a table advance step of 1 mm, a reconstruction increment of 1 mm (InnerEarSpi program Version A40, Siemens, Germany), a voltage of 120 kV, and a current of 70 mA. During visualization of the structures of the pyramid of the temporal bone, 2D images were used, both in the native axial planes and in the MPR reformation mode using a “bone” filter with a window width of 4000 μN and a window level of +700 μN. In addition, radiographic classifications of temporal bone anomalies were used [7,8,9,10].

### 4.4. Pathogenic Variant Analysis

Blood samples were collected from all 165 patients, and DNA was extracted from the blood leukocyte fraction using the phenol-chloroform method. In six of the 165 patients with incomplete partition of the cochlea (IP-1 and IP-2) in isolation or combined with an enlarged vestibular aqueduct (EVA) anomaly, we sequenced the coding region of the *SLC26A4*, *FOXI1*, and *KCNJ10* genes. To identify *SLC26A4* pathogenic variants, DNA fragments of the 21 exons of *SLC26A4*, including the flanking intronic regions, were sequenced using the oligonucleotide primers [2]. The entire coding regions of *FOXI1* and *KCNJ10* were also amplified and sequenced according to standard procedures, using the original primer sequences, as follows: *FOXI1*: 5’-CTGGGATCCACAGGCAGGT-3’ and 5’-AAGACTGGGGGATGCTACC-3’ for amplification of Exon 1 and 5’-TGCATCTGTCACCTTGGCTTT-3’ and 5’-CTGCGGAACTGCCCAGACAT-3’ for amplification of Exon 2; *KCNJ10*: (Part 1) 5’-GTCAGCTGGATTTCTACGATAACC-3’ and 5’-TTCCAAGTAGACACAGCCTCTG-3’, (Part 2) 5’-CCTCATGATCCGAGTTGCCAATA-3’ and 5’-ACTCACATTAGGAGGACCATGT-3’ for amplification of Exon 1. The PCR products of the variants’ elution profiles were sequenced using a Big Dye Terminator V1 kit (Applied Biosystems, Waltham, MA, USA) and ABI PRISM 3130XL (Applied Biosystems, USA) at the Genomics Core Facility, Institute of Chemical Biology and Fundamental Medicine, Siberian Branch of the Russian Academy of Sciences, Novosibirsk, Russia.

DNA sequence variations were identified by comparison with the reference sequences of the analyzed genes: *SLC26A4*: NC_000007.14, NG_008489.1, NM_000441.2, and NP_000432.1 (https://www.ncbi.nlm.nih.gov/gene/5172 accessed on 4 September 2021); *FOXI1*: NC_000005.10, NG_012068.2, NM_012188.5, and NP_036320.2 (https://www.ncbi.nlm.nih.gov/gene/2299 accessed on 4 September 2021); *KCNJ10*: NC_000001.11, NG_016411.1, NM_002241.5, and NP_002232.2 (https://www.ncbi.nlm.nih.gov/gene/3766 accessed on 4 September 2021). 

### 4.5. Database Searches

Population and phenotypic databases were used to search for previously described variants, namely dbSNP, a database for single nucleotide polymorphisms and other classes of minor genetic variation (https://www.ncbi.nlm.nih.gov/snp/) accessed on 4 September 2021; The Genome Aggregation Database (gnomAD), a resource developed by an international coalition of investigators, with the goal of aggregating and harmonizing both exome and genome sequencing data from a wide variety of large-scale sequencing projects (https://gnomad.broadinstitute.org/ accessed on 4 September 2021); The International Genome Sample (IGSR), a resource that maintains and shares the human genetic variation resources built by the 1000 Genomes Project (https://www.internationalgenome.org/ accessed on 4 September 2021); Online Mendelian Inheritance in Man (OMIM), an online catalog of human genes and genetic disorders containing a representative sample of disease-associated variants (https://www.omim.org/ accessed on 4 September 2021); The Human Gene Pathogenic variant Database (HGMD), a database of human gene pathogenic variants and gene damage responsible for human hereditary diseases (http://www.hgmd.cf.ac.uk/ac/index.php accessed on 4 September 2021); ClinVar, a freely accessible public archive of reports of the relationships among human variations and phenotypes, with supporting evidence (https://www.ncbi.nlm.nih.gov/clinvar/ accessed on 4 September 2022); and the Deafness Variation Database, which provides a comprehensive guide to the genetic variations in genes known to be associated with deafness (https://deafnessvariationdatabase.org/). These databases were accessed on 4 September 2022.

### 4.6. In Silico Analysis

The functional effects of the c.441G>A p.(Met147Ile) variant in the *SLC26A4* gene were predicted using SIFT (https://sift.bii.a-star.edu.sg/index.html) accessed on 2 February 2022 [62], PolyPhen-2 (http://genetics.bwh.harvard.edu/pph2) accessed on 2 February 2022 [63], Pathogenic variantTaster (https://www.pathogenic varianttaster.org/ accessed on 2 February 2022) [64], and PROVEAN (http://provean.jcvi.org/index.php) [65] (accessed on 23 May 2022). To model the spatial structure of the protein pendrin (SLC26A4), AlphaFold was used, which is a computational algorithm that can regularly predict protein structures with atomic accuracy, even in cases in which no similar structure is known [56]. AlphaFold produces a confidence metric for amino acid residues, as a predicted local distance difference test (pLDDT), on a scale of 0 to 100 [56]. An expected value of pLDDT > 90 is taken as the high accuracy cut-off (blue), pLDDT > 70 indicates low confidence and corresponds to a generally correct backbone prediction (turquoise color, good backbone prediction), pLDDT ≤ 70 indicates that we should also add substantial coverage for sequences without a good template in PDB (yellow color, should be considered with caution), and pLDDT < 50 indicates very low confidence (orange, should not be interpreted) [62]. Full details are available at: https://alphafold.ebi.ac.uk/about. accessed on 2 February 2022. Alignment of the mutant and normal structures of pendrin (SLC26A4) was carried out in the PyMol (Molecular Graphics System) graphics program, which provides 3D visualizations of proteins, small molecules, molecular surfaces, and trajectories [57]. Quantitative measurement of the similarity of the two spatial structures of proteins was carried out by calculating the root mean square deviation (RMSD) of the coordinates of the corresponding atomic positions, which is an indicator of the average distance between the atoms of superimposed proteins [66]. The criterion for complete similarity of the two structures was an RMSD value less than 2 Å [67,68]. Full details about the PyMol program are available at https://pymol.org/2/#products. Accessed on 15 February 2022.

## 5. Conclusions

1. The total contribution of biallelic *SLC26A4* cases among the patients with IP-1, IP-2, IP2+EVA, and isolated EVA anomalies was 66.7% (4 out 6 patients). In these cases, on the basis of the obtained clinical and molecular genetic data, DFNB4 was confirmed in three patients, and Pendred’s syndrome was confirmed in one patient. All monoallelic *SLC26A4* cases (2 out of 6 patients) were detected in patients of Siberian origin with the single-heterozygous variant c.757A>G p.(Ile253Val) (33.3%). Since, in this cohort of patients, we did not find a second mutant allele in this gene and did not find any causative variants in the *KCNJ10* and *FOXI1* genes, we supposed that in these patients, there are other pathogenic variants in the *trans*-position in the regulator region of this gene. In these cases, other extensive studies are required.

2. The morphology of the inner ear anomalies among patients with monoallelic *SLC26A4* cases demonstrated that IP-1 without EVA (50%) was the most common type of anomaly. Among patients with biallelic pathogenic variants in the *SLC26A4* gene, the predominant type of anomaly was IP-2+EVA (50.0%). However, for the first time, our study demonstrated that in biallelic *SLC26A4* patients all incomplete partitions of the cochlea are possible, from IP-1 and IP-2 to a normal cochlea. Considering that both types of cochlear anomalies are possible, radiographic differences between IP-1 and IP-2 cochlear anomalies in patients with biallelic *SLC26A4* pathogenic variants are significant for the surgical stage of cochlear implantation, since the IP-2 anomaly does not have an increased risk of “gushers” and recurrent meningitis, which is typical for IP-1 malformation.

**Limitations of the Study.** Although the strength of this study is the investigation of the three genes *SLC26A4*, FOXI1, and *KCNJ10* associated with DFNB4, it also has some limitations. The design of our study was based on current data of phenotype series and hypothesis of digenic inheritance of *SLC26A4* and *FOXI1* genes or *SLC26A4* and *KCNJ10* genes, annotated in the OMIM database (OMIM https://omim.org/entry/600791 accessed on 2 December 2022). We did not screen other autosomal genes, such as *GJB2* (three out of six patients in our sample had no changes in *GJB2* gene) (Table 2), *POU3F4*, *TMC1*, and *EPHA2* mentioned as associated with these phenotypes. In addition, since the CEVA-haplotype is associated with Caucasian genetic background, we could not test this hypothesis in *SLC26A4*-monoallelic patients of Siberian origin. However, we hope that further extensive studies of the *SLC26A4* monoallelic cases, among patients from similar isolated populations, will contribute to assessing the role of “silent” variants of this gene.

## Figures and Tables

**Table 1:** Allelic frequency and clinical significance of the *SLC26A4*, *FOXI1*, and *KCNJ10* gene variants identified in patients with IP-1, IP-2, IP-2+EVA, and isolated EVA.

Gene	Exon	Variant	dbSNP	Molecular Consequence	ClinVar Interpretation	Allelic Frequency **
*SLC26A4*	2	c.85G>C p.(Glu29Gln)	rs111033205	Missense	Pathogenic	10% (1/10)
	**5**	**c.441G>A p.(Met147Ile)**	**rs201905280**	**Missense**	**Conflicting interpretations of pathogenicity:** **uncertain significance(6), likely benign(1)**	**20%** **(2/10**)
	6	c.757A>G p.(Ile253Val)	rs773657545	Missense	Likely pathogenic *	20% (2/10)
*FOXI1*	17	c.2027T>A p.(Leu676Gln)	rs111033318	Missense	Pathogenic/likely pathogenic	20% (2/10)
	Intron 18	c.2089+1G>A (IVS18+1G>A)	rs727503430	Splice donor site	Pathogenic	10% (1/10)
	1	c.279G>A p.(Arg93=)	rs2277944	Synonymous	Benign	30% (3/10)
	2	c.1044T>C p.(Tyr348=)	rs10063424	Synonymous	Benign	90% (9/10)
*KCNJ10*	1	c.811C>T p.(Arg271Cys)	rs1130183	Missense	Benign/ likely benign	10% (1/10)

**Note**. The variant with uncertain significance (VUS) is highlighted in bold, * Data were taken from the Deafness Variation Database (DVD). ** One sibling was excluded from calculation of allelic frequency. In total, from six patients (12 chromosomes), the allelic frequency was calculated on five unrelated patients (10 chromosomes).

**Table 2:** Clinical and molecular genetic characteristics of patients with the studied inner ear malformations.

Patient Code	Age	Cipher	Sex	Ethnicity	Degree of HL	VD	Goiter/PDS	Side	Type of IP	EVA	DV	MM	*SLC26A4* Genotypes		*FOXI1* Genotypes		*KCNJ10* Genotypes	
													Allele 1	Allele 2	Allele 1	Allele 2	Allele 1	Allele 2
1095	2	-	M	Yakut	Severe	-	-/-	R	IP-1	-	+	-	**c.757A>G p.(Ile253Val)**	wt	c.279G>A p.(Arg93=)	c.1044T>C p.(Tyr348=)	wt	wt
								L	IP-1	+ (2.4 mm)	+	-						
18 *	52	-	F	Yakut	Profound	Dizziness	-/*-*	R	IP-1	-	+	-	**c.757A>G p.(Ile253Val)**	wt	c.279G>A p.(Arg93=) c.1044T>C p.(Tyr348=)	c.1044T>C p.(Tyr348=)	wt	wt
								L	IP-2	-	+	-						
16 *	38	II:3	F	Russian	Profound	Dizziness	-/-	R	IP-2	+ (3.5 mm)	+	+	**c.2089+1G>A** **(IVS18+1G>A)**	**c.85G>C** **p.(Glu29Gln)**	wt	wt	c.811C>T p.(Arg271Cys)	wt
								L	IP-2	+ (3.6 mm)	+	+						
17 *	32	II:4	F	Russian	Profound	-	-/-	R	IP-2	+ (2.9 mm)	+	+	**c.2089+1G>A** **(IVS18+1G>A)**	**c.85G>C** **p.(Glu29Gln)**	c.1044T>C p.(Tyr348=)	c.1044T>C p.(Tyr348=)	wt	wt
								L	IP-2	+ (2.7 mm)	+	+						
192	5	II:3	F	Russian	Severe	-	-/-	R	IP-1	+ (2.5 mm)	+	-	**c.441G>A** **p.(Met147Ile)**	**c.441G>A** **p.(Met147Ile)**	c.1044T>C p.(Tyr348=)	c.1044T>C p.(Tyr348=)		
								L	IP-1	-	+	-						
1091	33	II:3	F	Buryat	Moderate	-	Nodular goiter/PDS	R	Normal	+ (5.4 mm)	+	-	**c.2027T>A** **p.(Leu676Gln)**	**c.2027T>A p.(Leu676Gln)**	c.279G>A p.(Arg93=) c.1044T>C p.(Tyr348=)	c.1044T>C p.(Tyr348=)	wt	wt
								L	Normal	+ (5.7 mm)	+	-						

Note. HL, hearing loss; VD, vestibular dysfunctions; IP-1, incomplete partition Type 1 inner ear anomaly; IP-2, incomplete partition Type 2 inner ear anomaly [9,10]; EVA, enlarged vestibular aqueduct (greater than 1.5 mm in diameter) [8]; (-), vestibular dysfunctions not detected or information not available; PDS, Pendred syndrome; R, right; L, left; wt, wild type; DV, dilated vestibule; MM, Mondini malformation (IP-2 + a minimally dilated vestibule + an enlarged vestibular aqueduct) [9,10]. Pathogenic or likely pathogenic variants are highlighted in bold. *—DFNBA1-negative patients: pathogenic variants in *GJB2* gene and large deletions del(*GJB6*-D13S1830) and del(*GJB6*-D13S1854) were not found.

## Data Availability

The data presented in this study are available on request from the corresponding author.

## Supplementary Materials

The following supporting information can be downloaded at https://www.mdpi.com/article/10.3390/ijms232315372/s1.
