# Supplementary material for: Analysis of SLC26A4, FOXI1, and KCNJ10 Gene Variants in Patients with Incomplete Partition of the Cochlea and Enlarged Vestibular Aqueduct (EVA) Anomalies
Source: Int J Mol Sci. 2022 Dec 6;23(23):15372. doi: 10.3390/ijms232315372 (PMC9740095; doi:10.3390/ijms232315372)
Supplement: Supplementary file 1 [file ijms-23-15372-s001.zip › ijms-1952850-supplementary.pdf]

Supplementary

Analysis of *SLC26A4, FOXI1,* and *KCNJ10* Gene Variants in Patients with Incomplete Partition of the Cochlea and Enlarged Vestibular Aqueduct (EVA) Anomalies

Leonid A. Klarov [1,2,3], Vera G. Pshennikova [1,3], Georgii P. Romanov [1,3], Aleksandra M. Cherdonova [3], Aisen V. Solovyev [1,3], Fedor M. Teryutin [1,3], Nikolay V. Luginov [2,3], Petr M. Kotlyarov [4] and Nikolay A. Barashkov [1,3,*]

[1] Yakut Science Centre of Complex Medical Problems, 677000 Yakutsk, Russia
[2] Republican Hospital # 1—National Center of Medicine, 677019 Yakutsk, Russia
[3] Laboratory of Molecular Biology, M.K. Ammosov North-Eastern Federal University, 677027 Yakutsk, Russia
[4] Russian Scientific Center for Radiology, 117997 Moscow, Russia
* Correspondence: barashkov2004@mail.ru

Allelic frequency of *SLC26A4*, *FOXI1* and *KCNJ10* Genes Variants According to gnomAD

Table S1. The allelic frequency of the *SLC26A4*, *FOXI1* and *KCNJ10* genes variants according to gnomAD.

Gene	Variant	Exon	Latino/ Admixed American	Africa/ African American	European (Finnish)	European (non-Finnish)	Ashkenazi Jewish	East Asian	South Asian	Other	Total
SLC26A4	c.85G>C<br>p.(Glu29Gln)<br>rs111033205	2	0/26924<br>(0.000)	0/17794<br>(0.000)	2/13448<br>(0.0001487)	19/82994<br>(0.0002289)	0/8888<br>(0.000)	0/14046<br>(0.000)	0/23600<br>(0.000)	1/5694<br>(0.000)	22/193388<br>(0.0001138)
	c.441G>A<br>p.(Met147Ile)<br>rs201905280	5	0/35438<br>(0.000)	0/24970<br>(0.000)	129/25124<br>(0.005135)	20/129174<br>(0.0001548)	0/10368<br>(0.000)	4/19954<br>(0.0002311)	1/30616<br>(0.00003266)	7/7226<br>(0.0009687)	161/282870<br>(0.0005692)
	c.757A>G<br>p.(Ile253Val)<br>rs773657545	6	5/34592<br>(0.0001445)	1/16256<br>(0.00006152)	0/21646<br>(0.000)	0/113594<br>(0.000)	0/10074<br>(0.000)	11/18394<br>(0.0005980)	5/30616<br>(0.0001633)	0/6138<br>(0.000)	22/251310<br>(0.00008754)
	c.2027T>A<br>p.(Leu676Gln)<br>rs111033318	17	-	-	-	-	-	-	-	-	-
	c.2089+1G>A<br>(IVS18+1G>A)<br>rs727503430	Intron 18	0/339 20<br>(0.000)	5/23064<br>(0.0002168)	0/23782<br>(0.000)	1/109658<br>(0.000009119)	0/9594<br>(0.000)	0/19220<br>(0.000)	0/28240<br>(0.000)	0/6654<br>(0.000)	6/254132<br>(0.00002361)
FOXI1	c.279G>A<br>p.(Arg93=)<br>rs2277944	1	6558/29378<br>0.2232	10271/19070<br>(0.5386)	3991/20582<br>(0.1939)	15572/93884<br>(0.1659)	1745/9128<br>(0.1912)	7224/15196<br>(0.4754)	5810/25668<br>(0.2264)	1208/5964<br>(0.2025)	52379/21887<br>0<br>(0.2393)
	c.1044T>C<br>p.(Tyr348=)<br>rs10063424	2	32211/35170<br>0.9159	22306/24880<br>(0.8965)	21435/24860<br>(0.8622)	114114/126842<br>(0.8997)	9311/9974<br>(0.9335)	19651/19910<br>(0.9870)	27958/29948<br>(0.93360)	6397/7128<br>(0.8974)	253383/2787<br>12<br>(0.9091)
KCNJ10	c.811C>T<br>p.(Arg271Cys)<br>rs1130183	1	875/35440<br>(0.2469)	289/24956<br>(0.01158)	1965/25120<br>(0.07822)	9067/129090<br>(0.07024)	204/10364<br>(0.01968)	3/19954<br>(0.0001503)	452/30616<br>(0.01476)	341/7224<br>(0.04720)	13196/28276<br>4<br>(0.04667)

In Silico Assessment of the Possible Pathogenic Effect of the Missense Variant c.441G>A p.(Met147Ile) of the _SLC26A4_ Gene on the Function/Structure of the Pendrin (SLC26A4)

Evaluation of the c.441G>A p.(Met147Ile) variant of the SLC26A4 gene by predictive in silico tools

To predict the effect of the c.441G>A p.(Met147Ile) missense variant, we used following *in silico* tools: SIFT, Polyphen-2, PROVEAN, Mutation Taster. As a result, it was rated as "damaging" by all tools (Table S2).

Table S2. Evaluation of the c.441G>A p.(Met147Ile) variant of the *SLC26A4* gene by predictive *in silico* tools

Variant with uncertaine significance (VUS)	SIFT	Polyphen-2	PROVEAN	Mutation Taster
c.441G>A p.(Met147Ile)	Damaging Score: 0.000	Probably Damaging Score:1.000	Deleterious Score: -3.95	Disease causing

Modeling the structure of the pendrin (SLC26A4) using AlphaFold 2.0

Here, we propose a native of the three-dimensional (3D) structure of the SLC26A4 protein model based on the AlphaFold neural network architecture. According to a given amino acid (780 aa) sequence in the FASTA format: >ATG34016.1 SLC26A4 [Homo sapiens] (https://www.ncbi.nlm.nih.gov/protein/ATG34016.1?report=fasta). In Figure S1A, a general view of the predicted spatial structure of pendrin is presented. On the model, sections of the chain with different measure of reliability of predicting the amino acid sequence of the protein (pLDDT) are highlighted in different colors (https://alphafold.ebi.ac.uk/entry/O43511). The resulting model of pendrin, as a whole, has a relatively ordered structure and is predicted with high accuracy/corresponds to the correct prediction. However, "intrinsic disruption" is demonstrated by the N- and C-terminal regions, as well as a lone β-hairpin emerging from the membrane. These regions are orange/yellow, which corresponds to the very low reliability of the prediction of the AlphaFold algorithm and the true conformation in these regions may be different (Figure S1A). Similar to the structure of the known SLC26A5/SLC26Dg structures, the predicted SLC26A4 model consists of two components, a highly regular transmembrane domain (TMD) and a C-terminal cytoplasmic STAS domain (an acronym for "Sulfate Transporter Antagonist of anti-Sigma factor"), which is involved in the synthesis of various small molecules including nucleotides [4-8]. The amino acid (aa) residue Met147 analyzed by us in the predicted model is located in the α3-helix of the TMD and consists of 14 aa residues, from Pro142 to Met155. This section of the α3-helix has >90pLDDT>70, which indicates a high prediction accuracy (Figure S1B).

Comparative analysis of the sequences of amino acid residues that define transmembrane segments of human pendrin (SLC26A4) in accordance with the tertiary structural model obtained by the AlphaFold program with previously obtained TMD models of pendrin predicted on the basis of proteins homology to pendrin from the SLC26 family [2, 3, 5, 6], in the 3-segment, on which the missense p.(Met147Ile) substitution is located, a difference was found in the number of amino acid residues. On the resulting AlphaFold model, the α3-helix turned out to be represented by 14 aa residues from 142 to 155, in contrast to those previously described in other works, where that α3-helix TMD encompassed aa 141-147 in 70% of models, and 141-149 in 30% of models [2]. This information is important, since even a single missense mutation at a critical site on a helix can be detrimental to the folding and/or function of the protein [9].

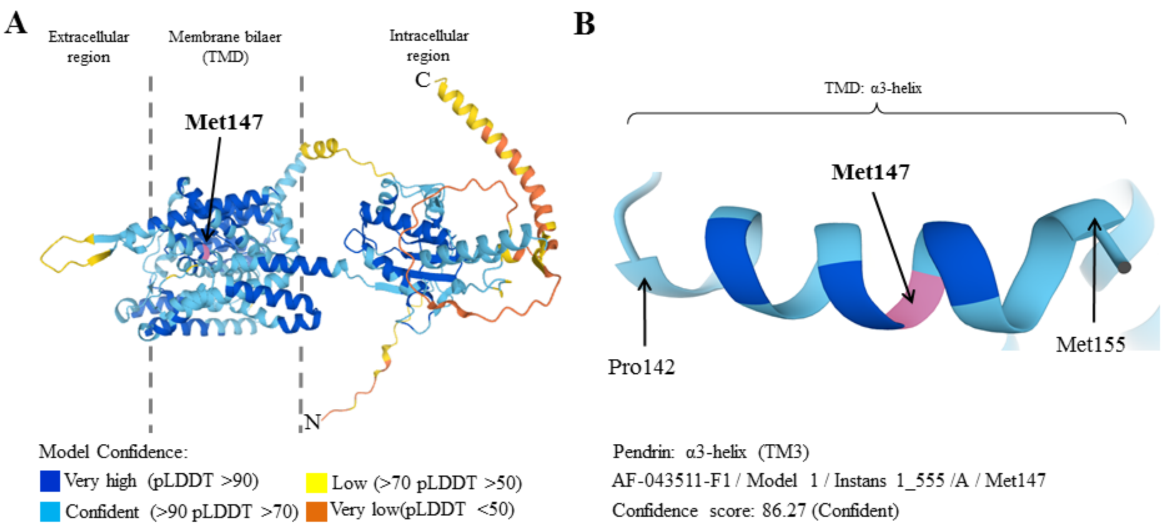

Figure S1. Three-dimensional (3D) spatial structural model of the human pendrin (SLC26A4) as predicted by Alphafold 2.0.

Note. (**A**) General view (https://alphafold.ebi.ac.uk/entry/O43511). Color represents regions of the monomer with varying confidence in conformation prediction (see bottom). Dashed lines indicate extra- and intracellular regions of the monomer. The arrows indicate the analyzed amino acid residues (Met147). (**B**) Close-up of the location of Met147 in the α3-helix of the TMD of pendrin (shown in pink).

Alignment of Mutant p.(Met147Ile) and Normal Structures of Pendrin (SLC26A4)

Using the PyMOL program tool based on the native structure of the SLC26A4 protein, modeled by the AlphaFold 2.0 program, we aligned the three-dimensional folding of the mutant and normal pendrin chain (Figure S2). For normal and mutant p.(Met147Ile) structures of pendrin, the calculated RMSD was 1.391 Å. The reflected differences in the folding of the N-, C-terminal sections of the chain in the compared forms of the protein (Figure S2A) are caused by the low reliability of the predictions of the conformation of this section by the AlphaFold algorithm and are ignored when calculating the RMSD index by the PyMOL program.

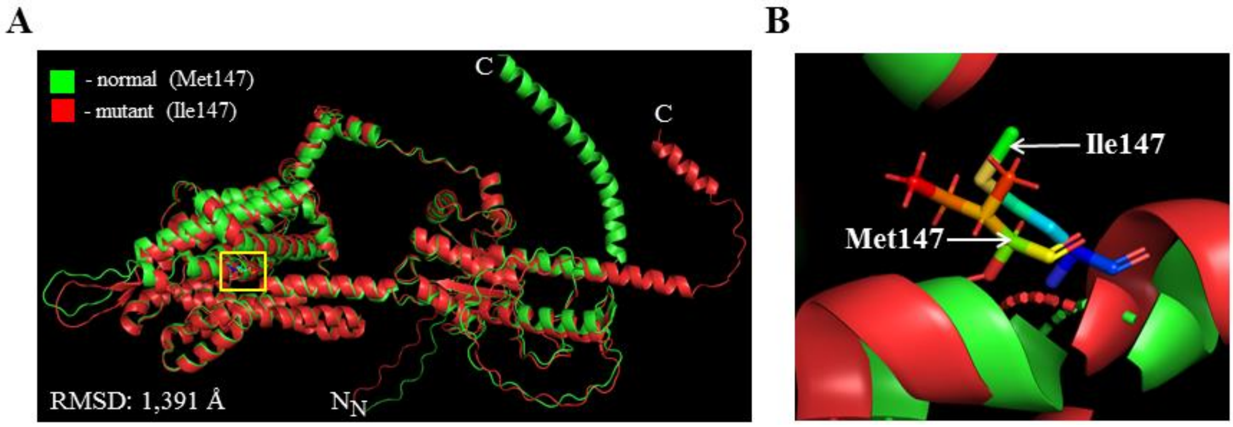

Figure S2. Alignment of mutant p.(Met147Ile) and normal structures of pendrin (SLC26A4) in PyMOL. Note. (**A**) The yellow square marks the localization site of the amino acid residue at position 147, in the norm Met (green), in the mutant Ile (red). (**B**) Close-up view of the side chains of the amino acid residues Met147 and Ile147, originating from the C-atoms of the main chain.

As a result of alignment of the three-dimensional folding of the mutant and normal chains using the PyMOL program, the obtained RMSD value: 1.391 Å is within the full similarity criterion (<2 Å) and indicates that the studied missense substitution c.441G>A p.(Met147Ile) of the *SLC26A4* gene does not

lead to a change in the spatial structure of the synthesized protein. This similarity of the two compared structures, with a slight difference, is probably due to the physicochemical properties of the considered amino acid residues in the polypeptide chain. It is known that the amino acids methionine and isoleucine belong to the same functional group of aliphatic amino acids with hydrophobic un-charged side radicals and their isoelectric point is approximately the same (Met: p*I* = 5.8, -COOH = 2.3, -NH3 = 9.2; Ile: p*I* = 6.1, -COOH = 2.4, -NH3 = 9.7).

Topology of the Pendrin Protein (SLC26A4)

However, the nature of the various mutational pathways cannot be fully explained by the physicochemical properties of the amino acid residues being replaced in the protein chain. Additional topological information of the protein must be used to understand and model the damaging factors, based on the location of the segments/motifs.

Based on the literature data on the structure of the SLC26 protein family and the data from UniProtKB: O43511 (https://www.uniprot.org/uniprot/O43511), we generated a schematic representation of the topology and structural motifs of SLC26A4 based on the model predicted by AlphaFold 2.0 (Figure S3).

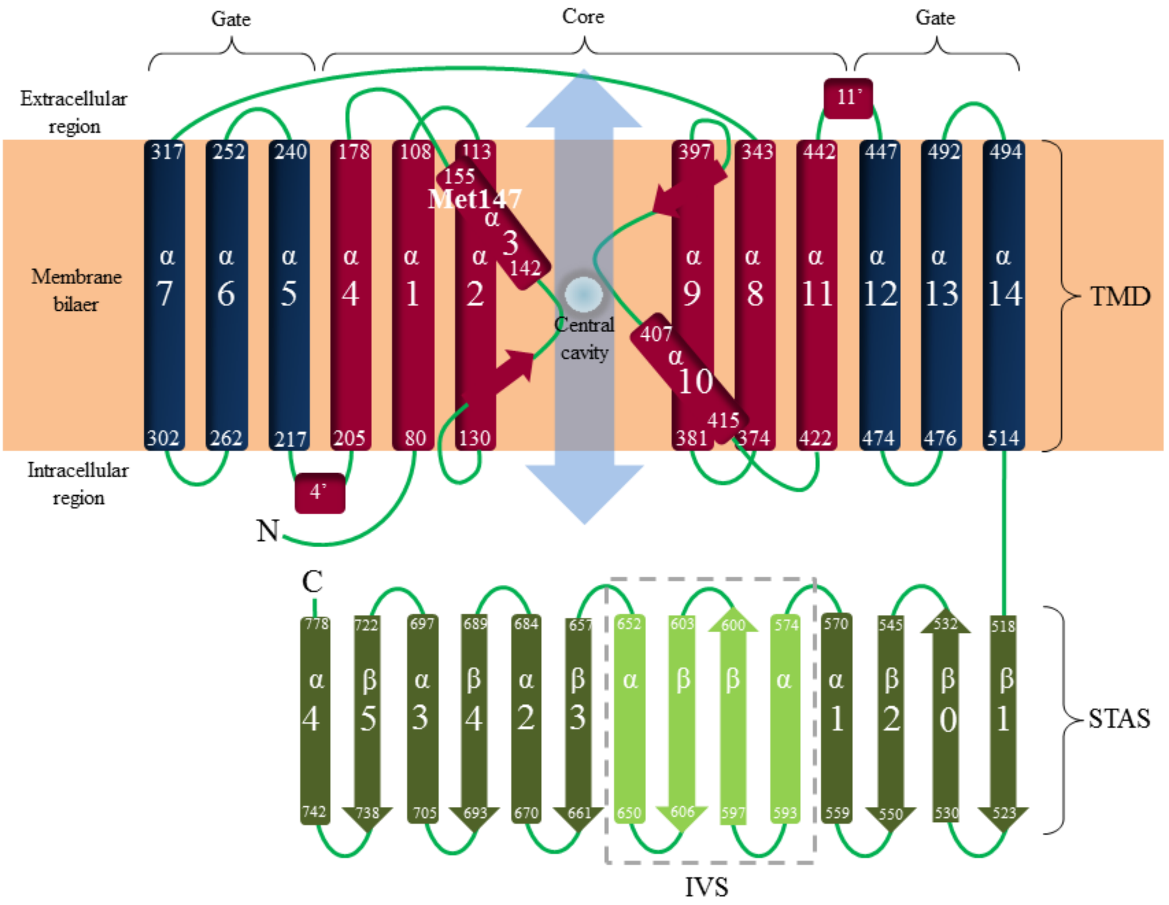

Figure S3. Topology of human SLC26A4 pendrin based on the model predicted by AlphaFold 2.0 [2, 5, 6].

Note. The transmembrane domain (TMD) consists of 14 TM-segments (α-helices) depicted as columns. The numbering of the segments is marked with large numbers in the center, along the edges there are amino acid positions in the α-helix sequence. The core domain (1-4 and 8-11) is marked in maroon. Gate domain (5-7 and 12-14), indicated in blue. In the center, the substrate (light sphere) is shown, where the path of its transportation passes. Below, in the cytoplasmic region, there is a packing scheme of the STAS-domain motif in the form of a "meander" (path of a protein chain), consisting of alternating β-strands and α-helices. The numbers indicate the order of the structural segments in the chain of the STAS domain.

Transmembrane domain (TMD) of pendrin is composed of 14 TM segments (α-helices) are arranged as two inverted repeat units, each consisting of 7 TM α-helices (1-7 and 8-14, their spans form the channel pores). Inner α-helices 1-4 and 8-11 pack to constitute the core domain, surrounded on one side by the gate domain consisting of outer α-helices 5-7 and 12-14 (Figure S3) [2, 5-8, 10]. In the core domain, not quite classical α-helices are two short α3 and α10 preceded by short β-strands, and the peculiar fold between them contributes to form a central cavity in the structure, where the substrate transport path lies [3]. The cytoplasmic region contains the N-terminal and C-terminal STAS domain consisting of alternating β-strands and α-helices (Figure S3). The STAS domain is typical of the SLC26 family of transporters, having a common structural fold consisting of 4-5 β-strands and 4 α-helices. However, a distinctive feature in mammals is the presence in the STAS domain of nominally structured intervening sequence (IVS), between the α1-helix and β3-strand, the function of which has not been fully studied (Figure S3) [7, 11]. Of note, AlphaFold long chain IVS pendrin was predicted with very low confidence (in Figure S1, orange chain with two yellow β-strands in the intracellular region).

In 2017 the work of Dossena et al. an important suggestion was made that the core domain is the key center of the ion channel, which include two transmembrane segments (3 and 10 α-helices) has a fundamental role for the recognition of different ions and substrate binding (Figure S4) [2].

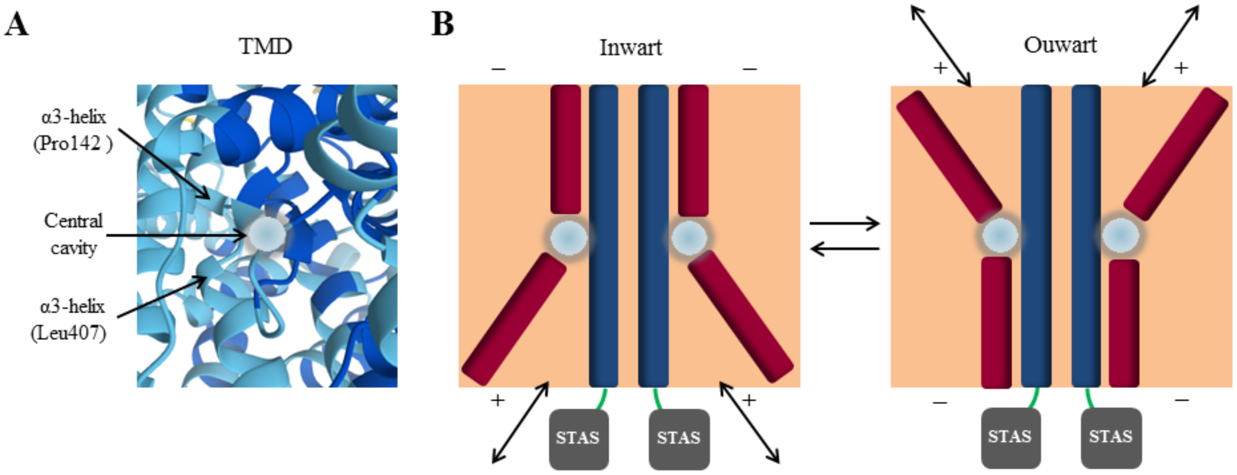

Figure S4. Schematic of the alternate access mechanism of anion transport in the core domain postulated for SLC26 transporter proteins [2, 6].

Note. (**A**) Fragment of the structural AlphaFold-model of pendrin, showing the central cavity in the transmembrane region. (**B**) Schematic of the access mechanism of anion transport in the core domain for SLC26 transporter proteins. The core domain (maroon) and gate domain (blue) regions of the TMD are embedded within the lipid bilayer (beige). The cytosolic STAS domain is represented as a dark square connected to the terminal helix of the gate domain. On the right, the bound anion substrate (light sphere) can be released to the cytoplasm from the inward facing conformation, or on the left, to the extracellular space from the outward facing conformation. The transition state between these events occurs in an occlusive (quasi-stable state) conformation, with the main domains facing both inward and outward simultaneously (not shown).

This translocation triggers conformational changes in the protein that ultimately alter its surface area in the plane of the plasma membrane. It swings anions across the membrane to get inside the cell, or vice versa, preventing them from dissociating and escape to the extracellular space (Figure S4B) [6, 11, 13, 14]. The cytosolic STAS domain probably acts as a competitive antagonist at the anion-binding site of the gate domain [2]. Thus, such an anion transport mechanism determines the common basis for the diverse functional behavior of the SLC26 family of transmembrane proteins [6]. Therefore, that packing of core domain helices α3 and α10 is crucial to integrity and conformational flexibility of the substrate translocation pathway [2]. It follows that mutant amino acid residues located in this part of the polypeptide chain can probably cause the pathophysiological mechanism of the SLC26A4 ion channel and cause diseases associated with the pendrin protein.

Analysis of Evolutionary Conservatism of Sequences α3-helix TM

For further estimation, we performed an evolutionary conservatism analysis of the α3-helix TM (Pro142 to Met155), which showed high conservation of aa positions 142 to 153 (Figure S5). The data was therefore obtained indicate that the c.441G>A p.(Met147Ile) mutation of the *SLC26A4* gene, which arose in an evolutionarily conserved region, can lead to the manifestation of diseases associated with a functional disruption of the core domain of the pendrin protein (SLC26A4).

TMD: α3-helix

FASTA: RevSeq	Organism	142	143	144	145	146	147	148	149	150	151	152	153	154	155
>ATG34016.1	[Homo sapiens]	P	V	V	S	L	M	V	G	S	V	V	L	S	M
>XP_019662624.2	[Ailuropoda melanoleuca]	P	V	V	S	L	M	V	G	S	V	V	L	S	M
>XP_014990157.2	[Macaca mulatta]	P	V	V	S	L	M	V	G	S	V	V	L	S	M
>XP_003407255.1	[Loxodonta africana]	P	V	V	S	L	M	V	G	S	V	V	L	N	M
>XP_005609131.2	[Equus caballus]	P	V	V	S	L	M	V	G	S	V	V	L	S	M
>XP_040116768.1	[Oryx dammah]	P	V	V	S	L	M	V	G	S	V	V	L	S	M
>XP_038310436.1	[Canis lupus familiaris]	P	V	V	S	L	M	V	G	S	V	V	L	S	M
>XP_036774867.1	[Manis pentadactyla]	P	V	V	S	L	M	V	G	S	V	V	L	S	L
>NP_062087.1	[Rattus norvegicus]	P	V	V	S	L	M	V	G	S	V	V	L	S	M
>NP_035997.1	[Mus musculus]	P	V	V	S	L	M	V	G	S	V	V	L	S	M
>XP_024598020.1	[Neophocaena asiaeorientalis asiaeorientalis]	P	V	V	S	L	M	V	G	S	V	V	L	S	M
		*	*	*	*	*	*	*	*	*	*	*	*	.	.

Figure S5. The result of alignment of 14 amino acid sequences localized in α3-helix TMD among vertebrate organisms.

Note. The α3-helix TMD amino acid sequence comparison (from Pro142 to Met155) was performed on a sample of pendrin protein homologues from 11 vertebrate organisms (Mammalia) obtained in the FASTA data format (https://www.ncbi.nlm.nih.gov/protein/?term =SLC26A4). As a result, the sequence was highly conserved starting from amino acid positions 142 to 153. The 147 amino acid position of the SLC26A4 protein is highlighted in red. * - conservative amino acid positions.

Missense Variants of the SLC26A4 Gene Leading to the Replacement of Methionine at 147 Amino Acid Position of the Pendrin

In addition, three more missense variants of the *SLC26A4* gene are known that lead to the replacement of methionine at amino acid position 147 of the pendrin: c.439A>C p.(Met147Leu), c.439A>G p.(Met147Val) and c.440T>C p.(Met147Thr). These variants have clinically confirmed pathogenic significance and are found mainly among European and Asian populations (Table S3). Accordingly with ACMG recommendation nucleotide changes in similar amino acid position have a strong pathogenic significant [1, 25]. The conflict in the interpretation of the pathogenicity of the c.441G>A p.(Met147Ile) variant is probably due to the fact that it was previously found only in the heterozygous state in patients with EVA [15, 16].

Table S3. Missense variants of the *SLC26A4* gene leading to the replacement of methionine at 147 amino acid position of the pendrin.

Missense replacement in 147 position of SLC26A4		dbSNP	Condition(s)	Clinical significance (ClinVar)	Allelic frequency in the world (gnomAD)		Reference
Nucleotide change	Protein change				Total	Highest occurrence	
c.439A>C	Leu	-	Not provided	Likely pathogenic (Aug 27, 2021)	-	-	https://www.ncbi.nlm.nih.gov/clinvar/variation/1067001/?new_evidence=true
c.439A>G	Val	rs760413427	Not provided, Autosomal recessive nonsyndromic hearing loss 4	Pathogenic (Sep 1, 2021)	0.000007953 (2 of 251464)	East Asia: 0.0001087 (2 of 18394)	[17-21]
c.440T>C	Thr	rs1554354787	Autosomal recessive nonsyndromic hearing loss 4, Pendred syndrome	Likely pathogenic (Feb 13, 2018)	-	-	[22-24]
**c.441G>A**	**Ile**	**rs201905280**	**Not provided, Not specified, Pendred syndrome, Autosomal recessive nonsyndromic hearing loss 4**	**Conflicting interpretations of pathogenicity (Nov 20, 2021)**	**0.0005692 (161 of 282870)**	**Europe (Finns): 0.005135 (129 of 25124)**	**[15, 16]**

Note. The c.441G>A p.(Met147Ile) variant in the *SLC26A4* gene with uncertain significance highlighted is bold.

Thus we concluded that the analyzed missense substitution p.Met147Ile of the pendrin protein (SLC26A4) theoretically does not violate the structural stability. To date, there is incomplete data on the causal relationship of the observed phenotypes with the mutation in question. However, if such a link is established, it is likely that the pathogenic effect of the p.(Met147Ile) mutation occurs at the functional level. This is due to the fact that p.(Met147Ile) is located in a critical region of the core domain (an evolutionarily conserved region of the TM α3-helix), disruption of which can lead to improper substrate transport or the appearance of toxic conformations.

Pathogenicity classification of the c.441G>A p.(Met147Ile) variant in the SLC26A4 gene, according to ACMG recommendations

As a result, the pathogenicity of the c.441G>A p.(Met147Ile) variant in the *SLC26A4* gene is classified in this work as "likely pathogenic", in accordance with the hereditary forms of hearing loss adapted criteria recommended by the ACMG [24, 25] (Table S4). The studied variant was previously described as variant with uncertain significance (VUS).

Table S4. Pathogenicity criteria of the c.441G>A p.(Met147Ile) variant in the *SLC26A4* gene, according to ACMG with HL-EP Specifications [1, 25].

Pathogenic criteria		The c.441G>A p.(Met147Ile) variant in the *SLC26A4* gene
Strong	**PM5** Missense change at same codon as two different pathogenic missense variants	There are three known pathogenic missense variants at 147th amino acid position of the SLC26A4 protein [Table S3]
Supporting	**PM2** Low MAF in population databases <0.0007 (0.07%) for autosomal recessive diseases	The frequency of the c.441G>A p.(Met147Ile) variant in the *SLC26A4* gene in gmomAD database is 161/282870 (0.0005692) (<0.07%) [Table S1]
	**PP2** Missense variant in a gene that has a low rate of benign missense variation and in which missense variants are a common mechanism of disease	The *SLC26A4* gene has been characterized as a gene that has a low level of benign missense variations and missense variants are common mechanism of disease in this gene [25]
	**PP3** Multiple lines of computational evidence support a deleterious effect on the gene or gene product	Deleterious effect was shown by four pathogenicity prediction tools (SIFT, Polyphen-2, PROVEAN, Mutation Taster) (Table S2) + other *in silico* evidence [Chapter 2]
Interpretation	1 Strong (**PM5**) + 3 Supporting (**PM2+PP2+PP3**)	Likely pathogenic

Supplementary References

1. Richards, S.; Aziz, N.; Bale, S.; Bick, D.; Das, S.; Gastier-Foster, J.; Grody, W.W.; Hegde, M.; Lyon, E.; Spector; E. et al. ACMG Laboratory Quality Assurance Committee. Standards and guidelines for the interpretation of sequence variants: a joint consensus recommendation of the American College of Medical Genetics and Genomics and the Association for Molecular Pathology. Genet Med. 2015, 17, 405-24. doi: 10.1038/gim.2015.30.
2. Dossena, S.; Bernardinelli, E.; Sharma, A.K.; Alper, S.L.; Paulmichlet, M. The Pendrin Polypeptide. In: Dossena, S., Paulmichl, M. (eds) *The Role of Pendrin in Health and Disease. Springer, Cham* **2017**, 187-220, https://doi.org/10.1007/978-3-319-43287-8_11.
3. Bassot, C.; Minervini, G.; Leonardi, E.; Tosatto, S.C.E. Mapping pathogenic mutations suggests an innovative structural model for the pendrin (SLC26A4) transmembrane domain. *Biochimie* **2017**, 132, 109-120, doi: 10.1016/j.biochi.2016.10.002.
4. Babu, M.; Greenblatt, J.F.; Emili, A.; Strynadka, N.C.J.; Reithmeier, R.A.F.; Moraes, T.F. Structure of a SLC26 Anion Transporter STAS Domain in Complex with Acyl Carrier Protein: Implications for E. Coli YchM in Fatty Acid Metabolism. *Structure* **2010**, 18, 1450–1462, doi:10.1016/j.str.2010.08.015.
5. Gorbunov, D.; Sturlese, M.; Nies, F.; Kluge, M.; Bellanda, M.; Battistutta, R.; Oliver, D. Molecular architecture and the structural basis for anion interaction in prestin and SLC26 transporters. *Nat Commun* **2014**, 5, 3622, doi: 10.1038/ncomms4622.
6. Geertsma, E.R.; Chang, Y.-N.; Shaik, F.R.; Neldner, Y.; Pardon, E.; Steyaert, J.; Dutzler, R. Structure of a Prokaryotic Fumarate Transporter Reveals the Architecture of the SLC26 Family. *Nat Struct Mol Biol* **2015**, 22, 803–808, doi:10.1038/nsmb.3091.
7. Sharma, A.K.; Krieger, T.; Rigby, A.C.; Zelikovic, I.; Alper, SL. Human SLC26A4/Pendrin STAS domain is a nucleotide-binding protein: Refolding and characterization for structural studies. *Biochem Biophys Rep* **2016**, 8, 184-191, doi: 10.1016/j.bbrep.2016.08.022.
8. Kuwabara, M.F.; Wasano, K.; Takahashi, S.; Bodner, J.; Komori, T.; Uemura, S.; Zheng, J.; Shima, T.; Homma, K. The Extracellular Loop of Pendrin and Prestin Modulates Their Voltage-Sensing Property. *J Biol Chem* **2018**, 293, 9970–9980, doi:10.1074/jbc.RA118.001831.
9. Ng, D.P.; Poulsen, B.E.; Deber, C.M. Membrane protein misassembly in disease. *Biochimica et Biophysica Acta (BBA)-Biomembranes* **2012**, 1818(4), 1115–22, doi: 10.1016/j.bbamem.2011.07.046.
10. Rapp, C.; Reinhart, X.B.; Reithmeier, A.F. Molecular analysis of human solute carrier SLC26 anion transporter disease-causing mutations using 3-dimensional homology modeling. *Biochim Biophys Acta Biomembr* **2017**, 1859, 2420-2434, doi: 10.1016/j.bbamem.2017.09.016.
11. Pasqualetto, E.; Aiello, R.; Gesiot, L.; Bonetto, G.; Bellanda, M. Battistutta, R. Structure of the cytosolic portion of the motor protein prestin and functional role of the STAS domain in SLC26/SulP anion transporters. *J Mol Bio* **2010**, 400, 448-462, doi: 10.1016/j.jmb.2010.05.013.
12. Sharma, A.K.; Rigby, A.C.; Alper, S.L. STAS domain structure and function. *Cell Physiol Biochem* **2011**, 28, 407-22, doi: 10.1159/000335104.
13. Detro-Dassen, S.; Schänzler, M.; Lauks, H.; Martin, I.; zu Berstenhorst, S.M.; Nothmann, D.; Torres-Salazar, D.; Hidalgo, P.; Schmalzing, G.; Fahlke, C. Conserved Dimeric Subunit Stoichiometry of SLC26 Multifunctional Anion Exchangers. *J Biol Chem* **2008**, 283, 4177–4188, doi:10.1074/jbc.M704924200.
14. Farrell, B.; Skidmore, B.L.; Rajasekharan, V.; Brownell, W.E. A novel theoretical framework reveals more than one voltage-sensing pathway in the lateral membrane of outer hair cells. *J Biol Che*m **2020**, 152(7):e201912447, doi: 10.1085/jgp.201912447.
15. Jonard, L.; Niasme-Grare, M.; Bonnet, C.; Feldmann, D.; Rouillon, I.; Loundon, N.; Calais, C.; Catros, H.; David, A.; Dollfus, H.; et al. Screening of SLC26A4, FOXI1 and KCNJ10 Genes in Unilateral Hearing Impairment with Ipsilateral Enlarged Vestibular Aqueduct. *Int J Pediatr Otorhinolaryngol* **2010**, 74, 1049–1053, doi:10.1016/j.ijporl.2010.06.002.

16. Fu, C.; Zheng, H.; Zhang, S.; Chen, Y.; Su, J.; Wang, J.; Xie, B.; Hu, X.; Fan, X.; Luo, J.; et al. Mutation screening of the SLC26A4 gene in a cohort of 192 Chinese patients with congenital hypothyroidism. *Arch Endocrinol Metab* **2016**, 60, 323-7, doi: 10.1590/2359-3997000000108.
17. Park, H.J.; Lee, S.J.; Jin, H.S.; Lee, J.O.; Go, S.-H.; Jang, H.S.; Moon, S.-K.; Lee, S.-C.; Chun, Y.-M.; Lee, H-K.; et al. Genetic basis of hearing loss associated with enlarged vestibular aqueducts in Koreans. Clin Genet **2005**, 67, 160–165, https://doi.org/10.1111/j.1399-0004.2004.00386.x
18. Tsukamoto, K.; Suzuki, H.; Harada, D.; Namba, A.; Abe, S.; Usami, S. Distribution and Frequencies of PDS (SLC26A4) Mutations in Pendred Syndrome and Nonsyndromic Hearing Loss Associated with Enlarged Vestibular Aqueduct: A Unique Spectrum of Mutations in Japanese. Eur J Hum Genet 2003, 11, 916–922, doi:10.1038/sj.ejhg.5201073.
19. Yoon, J.S.; Park, H.-J.; Yoo, S.-Y.; Namkung, W.; Jo, M.J.; Koo, S.K.; Park, H.Y.; Lee, W.S.; Kim, K.H.; Lee, M.G. Heterogeneity in the processing defect of SLC26A4 mutants. *J Med Genet* **2008**, 45, 411-9, doi: 10.1136/jmg.2007.054635.
20. Ishihara, K.; Okuyama, S.; Kumano, S.; Iida, K.; Hamana, H.; Murakoshi, M.; Kobayashi, T.; Usami, S.; Ikeda, K.; Haga, Y.; et al. Salicylate Restores Transport Function and Anion Exchanger Activity of Missense Pendrin Mutations. *Hear Res* **2010**, 270, 110–118, doi:10.1016/j.heares.2010.08.015.
21. Huang, S.; Han, D.; Yuan, Y.; Wang, G.; Kang, D.; Zhang, X.; Yan, X.; Meng, X.; Dong, M.; Dai, P. Extremely discrepant mutation spectrum of SLC26A4 between Chinese patients with isolated Mondini deformity and enlarged vestibular aqueduct. *J Transl Med* **2011**, 9, 167, doi: 10.1186/1479-5876-9-167.
22. Albert, S.; Blons, H.; Jonard, L.; Feldmann, D.; Chauvin, P.; Loundon, N.; Sergent-Allaoui, A.; Houang, M.; Joannard, A.; Schmerber, S.; et al. SLC26A4 Gene Is Frequently Involved in Nonsyndromic Hearing Impairment with Enlarged Vestibular Aqueduct in Caucasian Populations. *Eur J Hum Genet* **2006**, 14, 773–779, doi:10.1038/sj.ejhg.5201611.
23. Rebeh, I.B.; Yoshimi, N.; Hadj-Kacem, H.; Yanohco, S.; Hammami, B.; Mnif, M.; Araki, M.; Ghorbel, A.; Ayadi, H.; Masmoudi, S.; Miyazaki, H. Two missense mutations in SLC26A4 gene: a molecular and functional study. Clin Genet 2010, 78, 74-80, doi: 10.1111/j.1399-0004.2009.01360.x.
24. Wasano, K.; Takahashi, S.; Rosenberg, S.K.; Kojima, T.; Mutai, H.; Matsunaga, T.; Ogawa, K.; Homma, K. Systematic Quantification of the Anion Transport Function of Pendrin (SLC26A4) and Its Disease-Associated Variants. *Hum Mutat* **2020**, 41, 316–331, doi:10.1002/humu.23930.
25. Oza, A.M.; DiStefano, M.T.; Hemphill, S.E.; Cushman, B.J.; Grant, A.R.; Siegert, R.K.; Shen, J.; Chapin, A.; Boczek, N.J.; Schimmenti, L.A.; et al. ClinGen Hearing Loss Clinical Domain Working Group. Expert specification of the ACMG/AMP variant interpretation guidelines for genetic hearing loss. *Hum Mutat* **2018,** 39, 1593-1613. doi: 10.1002/humu.23630.
